# The behavioural response of mice lacking NK_1_ receptors to guanfacine resembles its clinical profile in treatment of ADHD

**DOI:** 10.1111/bph.12860

**Published:** 2014-09-26

**Authors:** Katharine Pillidge, Ashley J Porter, Julia A Dudley, Yuan-Chen Tsai, David J Heal, S Clare Stanford

**Affiliations:** 1 Department of Neuroscience, Physiology and Pharmacology, University College LondonLondon, UK; 2 Department of Cell and Developmental Biology, University College LondonLondon, UK; 3 RenaSci Ltd.BioCity, Nottingham, UK

## Abstract

**Background and Purpose:**

Mice with functional ablation of substance P-preferring neurokinin-1 receptors (NK1R−/− mice) display behavioural abnormalities resembling those in attention deficit hyperactivity disorder (ADHD). Here, we investigated whether the ADHD treatment, guanfacine, alleviated the hyperactivity and impulsivity/inattention displayed by NK1R−/− mice in the light/dark exploration box (LDEB) and 5-choice serial reaction–time task (5-CSRTT), respectively. Following reports of co-morbid anxiety in ADHD, we also investigated effects of guanfacine on anxiety-like behaviour displayed by NK1R−/− and wild-type (WT) mice in the elevated plus maze (EPM).

**Experimental Approach:**

Mice were treated with guanfacine (0.1, 0.3 or 1.0 mg·kg^−1^, i.p.), vehicle or no injection and tested in the 5-CSRTT or the LDEB. Only the lowest dose of guanfacine was used in the EPM assays.

**Key Results:**

In the 5-CSRTT, a low dose of guanfacine (0.1 mg·kg^−1^) increased attention in NK1R−/− mice, but not in WT mice. This dose did not affect the total number of trials completed, latencies to respond or locomotor activity in the LDEB. Impulsivity was decreased by the high dose (1.0 mg·kg^−1^) of guanfacine, but this was evident in both genotypes and is likely to be secondary to a generalized blunting of behaviour. Although the NK1R−/− mice displayed marked anxiety-like behaviour, guanfacine did not affect the behaviour of either genotype in the EPM.

**Conclusions and Implications:**

This evidence that guanfacine improves attention at a dose that did not affect arousal or emotionality supports our proposal that NK1R−/− mice express an attention deficit resembling that of ADHD patients.

**Linked Articles:**

This article is part of a themed section on Animal Models in Psychiatry Research. To view the other articles in this section visit http://dx.doi.org/10.1111/bph.2014.171.issue-20

## Tables of Links

**Table:** 

TARGETS	LIGANDS	
α2A-adrenoceptors	d-Amphetamine	Methylphenidate
NK1 receptors	Guanfacine	RP 67580

These Tables list key protein targets and ligands in this article which are hyperlinked to corresponding entries in http://www.guidetopharmacology.org, the common portal for data from the IUPHAR/BPS Guide to PHARMACOLOGY (Pawson *et al*., 2014) and are permanently archived in the Concise Guide to PHARMACOLOGY 2013/14 (Alexander *et al*., 2013).

## Introduction

Mice from a 129/Sv × C57BL/6J genetic background (crossed with an outbred MF1 strain), with functional ablation of the substance P-preferring, neurokinin-1 (NK_1_) receptor gene (referred to as NK1R−/−mice) (de Felipe *et al*., 1998), express locomotor hyperactivity in an activity meter (Herpfer *et al*., 2005) and the light/dark exploration box (LDEB) (Herpfer *et al*., 2005; Fisher *et al*., 2007; Yan *et al*., 2010). They also display deficits in cognitive performance and response control when tested in the 5-choice serial reaction–time task (5-CSRTT). Specifically, when compared with their wild-type (WT) counterparts, they typically score greater *%omissions* (an index of inattentiveness) and increased *%premature responses* (an index of motor impulsivity) in this test (Yan *et al*., 2011; Dudley *et al*., 2013). Mechanism(s) that could explain how a lack of functional NK_1_ receptors could provoke locomotor hyperactivity, inattentiveness and impulsivity are reviewed in Yan *et al*. (2010) and Stanford (2014).

These abnormal behaviours (hyperactivity, inattention and impulsivity) resemble the core diagnostic features of attention deficit hyperactivity disorder (ADHD). Our proposal that NK1R−/− mice can be used to study the behavioural and cognitive abnormalities seen in ADHD patients is supported by evidence from translational studies, which discovered that an association between polymorphisms in, or near, the human equivalent of the NK_1_ receptor gene (*TACR1*) predicts increased vulnerability to ADHD (Sharp *et al*., 2009; 2014; Yan *et al*., 2010).

Psychomotor stimulants (*d*-amphetamine, methylphenidate and the prodrug, lisdexamfetamine) are the first-line treatments for ADHD. Only two other compounds are licensed for this clinical indication: the preferential noradrenaline reuptake inhibitor, atomoxetine (Preti, 2002), and the α_2A_-adrenoceptor agonist, guanfacine (Biederman *et al*., 2008; Sallee *et al*., 2009). An extended release formulation of guanfacine was approved for the treatment of ADHD in the United States in 2009.

We have reported previously that the *hyperactivity* of NK1R−/− mice is attenuated by the psychostimulants, *d*-amphetamine and methylphenidate, as in ADHD (Yan *et al*., 2009; 2010), but *d*-amphetamine did not prevent impulsivity or inattentiveness in the 5-CSRTT (Yan *et al*., 2011). This could be relevant to reports that *d*-amphetamine is ineffective in about 25% of ADHD patients (Heal *et al*., 2009). The first objective of this study was to investigate whether guanfacine ameliorates the deficits in cognitive performance or response control that are expressed by NK1R−/− mice in the 5-CSRTT, in line with its efficacy in treating ADHD.

A second objective was to establish whether or not guanfacine prevents the locomotor hyperactivity of NK1R−/− mice. It has already been reported that this drug reduces locomotor activity of the SHR (an established rodent model of ADHD) in the open field, but not that of their control strains (Wistar or Wistar Kyoto; Langen and Dost, 2011). However, we were mindful of the problem that animals’ emotional status (anxiety-like behaviour) can confound measures of locomotor activity in this test (see Stanford, 2007a,b; Wilcock and Broadhurst, 1967). This is especially important for studies of the effects of guanfacine on motor behaviour because this drug is used to treat anxiety, which is a common co-morbid disorder in ADHD (Sobanski, 2006). For that reason, we also compared the effects of guanfacine on the behaviour of NK1R−/− mice and WT in the elevated plus maze (EPM), an established screen for anxiolytic drugs.

A final caveat is that, although NK1R−/− mice display *inattention* and *impulsivity* when they derive from separate homozygous, inbred strains (‘homs’), the behavioural phenotype of homozygous progeny from heterozygote NK1R+/− (F1) breeding pairs (‘*hets*’) is as yet unknown. This, together with growing interest in epigenetic influences in ADHD, prompted us to investigate whether any effects of guanfacine on behaviour in the 5-CSRTT differ in WT and NK1R−/− mice derived from these two breeding methods.

The findings from these studies lead us to infer that a low dose of guanfacine (0.1 mg·kg^−1^) reduces inattentiveness of NK1R−/− mice in the 5-CSRTT, but that higher doses (10 mg·kg^−1^) reduce impulsivity in both genotypes. The latter response to guanfacine is likely to be a consequence of the sedative effects of this drug. Neither of these responses to guanfacine is dependent on the breeding method nor is likely to be explained by any change in animals’ emotional status.

## Methods

### Animals

All animal care and experimental procedures complied with the Animals (Scientific Procedures) Act, 1986 (UK) and were approved by the Ethical Review Panel at University College London. These studies comply with the ARRIVE guidelines for reporting experiments involving animals (Kilkenny *et al*., 2010; McGrath *et al*., 2010). A total of 104 animals were used in these experiments (24 in the 5-CSRTT, 50 in the LDEB and 30 in the EPM).

Mouse colonies were bred at University College London in a facility held at 21 ± 2°C, 45 ± 5% humidity, with a 12:12 h light: dark cycle (lighting increased in steps from 07:00 to 08:00 h and reduced in steps from 19:00 to 20:00 h). The home cages incorporated environmental enrichment and were cleaned twice weekly (bedding obtained from Litaspen Premium, Lillico, Horley, Surrey, UK). Food supply was obtained from Harlan (Bicester, UK: 2018 global rodent diet).

All mice were bred from the same background strain (129/Sv × C57BL/6J crossed with an outbred MF1 strain, more than 10 generations ago) (de Felipe *et al*., 1998). Half of the mice used in the 5-CSRTT experiment comprised homozygous WT (NK1R+/+) and NK1R−/− mice from inbred homozygous parents (*homs*) (*N* = 6 per group). The subjects were the progeny from two breeding pairs for each genotype. The remainder, which were tested at the same time as the *homs*, comprised (F2) homozygous WT and NK1R−/− mice derived from three breeding pairs of heterozygous (NK1R+/−) parents, which were the (F1) progeny of inbred WT (NK1R+/+) mice crossed with inbred NK1R−/− mice (WT *hets* and NK1R−/− *hets*) (*N* = 6 per group).

Each home cage contained two to four mice. The wild-type and NK1R−/− *homs* were housed separately, but *het* mice were housed in cages that contained at least one WT and one NK1R−/− mouse. Only mice from homozygous parents (inbred strains) were used in the LDEB and EPM because there were no differences in the effects of guanfacine on the behaviour of the two colonies of mice in the 5-CSRTT.

### 5-CSRTT

Twelve WT male and 12 NK1R−/− male mice were used at 6–8 weeks of age (weighing WT *hom*: 30.5–35.6 g; NK1R−/− *hom*: 26.7–30.9 g; WT *het*: 31.1–41.1 g; NK1R−/− *het*: 30.5–34.7 g at the start of training). The mice were brought into the laboratory every day (Monday to Friday) between 09:00 and 09:30 h and weighed before training/testing in the 5-CSRTT, which took place between 10:00 and 12:00 h (AM session) or 13:00 and 15:00 h (PM session). The animals were returned to the holding room at 16:00 h and each cage supplied with a weighed quota of food, which was adjusted to stabilize subjects at 90% of their free-feeding body weight. Water was freely available at all times. One WT *het* and one NK1R−/− *het* failed to graduate through the training phase of the procedure and were withdrawn from the study.

The apparatus, supplied by Med Associates (St. Albans, VT, USA), was controlled by a Smart Ctrl Package 8IN/16OUT with an additional interface by MED-PC for Windows (Med Associates). This comprised four sound-attenuated operant chambers with five equally spaced apertures, incorporated into one wall of each chamber: these apertures could be illuminated independently. A nose-poke by the mice into each hole was scored following interruption of an infrared beam that spanned the hole. Interceptions of an infrared beam across a magazine in the opposite wall, which delivered the reward (0.01 mL of 30% condensed milk solution), were also scored: these occurred whenever a mouse collected the reward and initiated the next ‘trial’. Incorrect, omitted and premature responses were punished with a 5 s time out, during which the house light was extinguished and no new trials could be initiated. Perseverative responses were not punished.

The procedure was the same as that previously reported and is documented in full elsewhere (Yan *et al*., 2011). Briefly, mice were assigned to one of four test chambers in a fully counterbalanced design and were tested in the same chamber throughout. First, they were habituated to the apparatus for 3 days and then trained in the 5-CSRTT, graduating through increasingly challenging stages (1–6) after fulfilling the criteria for each stage (see Yan *et al*., 2011). The final stimulus duration (SD) at stage 6 was 1.8 s. After reaching the criteria for a stable baseline at stage 6 (total trials completed minus premature responses = 100; >75% accuracy; <25% omissions), for at least 3 consecutive days, the mice were eligible for testing with a variable inter-trial interval [VITI; 2, 5, 10 or 15 s (delivered on a random schedule) with an SD of 1.8 s]. Mice were tested with the VITI once weekly, on Fridays. A VITI test was used because it prevents the use of interval timing as a strategy for correct responding, thereby increasing cognitive load. The first week of testing was carried out with treatment–naïve mice [no injection (1): ‘NI-1′] only (reported elsewhere). In each of the following 5 weeks, mice were subject to a VITI test, 30 min after an i.p. injection of either vehicle (saline, 10 mL·kg^−1^) or guanfacine (0.1, 0.3 or 1.0 mg·kg^−1^), or a second untreated session [no injection (2): ‘NI-2′]. These drug doses were chosen because they modify relevant aspects of behaviour in mice: for example, working memory in a T-maze (Franowicz *et al*., 2002) and locomotor activity (Archer and Fredriksson, 2003). NI-2 (baseline behaviour of uninjected mice) was embedded within the sequence of vehicle/drug treatments to control for any systematic changes in behaviour arising from rehearsal of the test (see Weir *et al*., 2014). The vehicle/drug/NI-2 sessions were counterbalanced across subjects, using a pseudo William’s Latin square, such that each mouse received each treatment, or NI-2 session, once only.

The test sessions were terminated after either 45 min or after the mouse completed 100 trials, plus the number of premature responses, whichever occurred first. Performance scores in the 5-CSRTT were recorded and stored online (see Table 1). *Omissions* and *premature responses* were calculated per 100 trials to correct for any differences in the total number of trials completed by the mice.

**Table 1:** Performance variables recorded in the testing phase of the 5-CSRTT

Behavioural outcome	Method of calculation
Total number of trials completed	Total correct responses + total incorrect responses + total omissions
%Accuracy	[correct responses/(correct + incorrect responses)] × 100
%Omissions	[total omissions/total number of trials] × 100
%Premature responses	[premature responses/(total number of trials + prems)] × 100
Latency to correct response	Duration between onset of stimulus and a nose-poke in the correct hole
Latency to collect the reward	Duration between a nose-poke in the correct hole and collection of reward from the magazine
Perseveration	Number of unnecessary responses into the correct hole after the initial correct response, before collection of reward, per 100 trials

### LDEB

A separate cohort of NK1R−/− and WT mice, from homozygous breeding pairs, was tested in the LDEB, which was dimly lit [dark zone (DZ): 4 lux, light zone (LZ): 20 lux]. One WT and one NK1R−/− mouse were tested simultaneously, with the same treatment and in adjacent LDEBs, to balance any nuisance factors across the two genotypes. The procedure is described fully in Fisher *et al*. (2007) and Herpfer *et al*. (2005). Briefly, mice were allowed to habituate to the test room between 10:00 and 13:00 h. At either 13:00 or 15:30 h, they were confined individually within the DZ of the LDEB for 60 min, after which they were injected with their allocated treatment, or left untreated (no injection, NI), and replaced in the DZ for a further 30 min. The treatments were either vehicle (0.9% saline, 10 mL·kg^−1^) or guanfacine (0.1, 0.3 or 1.0 mg·kg^−1^, i.p.) (*N* = 5 per group), which were given in a counterbalanced sequence; each mouse received only one treatment. After a total of 90 min in the DZ, the mice were transferred to the LZ and allowed to move freely between the two zones. Behaviour was recorded by a digital video camera for 30 min and scored later by an observer, unaware of the treatments. Because the activity of mice in the LDEB declined progressively to reach a ‘floor’ after approximately 15 min, only the first 10 min of activity after transfer to the LZ were used in the statistical analysis.

### EPM

A third cohort of mice (from homozygous breeding pairs, only) was tested in the EPM. Mice were allowed to habituate to the test room between 10:00 and 14:00 h, and tested between 14:00 and 16:00 h on the EPM. They then received an i.p. injection of vehicle (saline) or guanfacine (0.1 mg·kg^−1^), 30 min before testing, or received no injection (*N* = 5 per group). Treatments were assigned in a counterbalanced order. The mice were tested individually, by placing them at the centre of the four arms, facing an open arm, after which they were allowed to explore the maze for 5 min. The maze was cleaned between each test with 70% ethanol. Behaviour was recorded by a video camera, positioned above the maze and was scored later by an observer, unaware of the treatments. The following behavioural measures were recorded:

- %Time in open arms [time with all four paws in open arms/(time on open arms + time on closed arms)] × 100
- %Time in centre; (time in centre/total time) × 100
- Number of whole-body entries; all four paws enter the arm

### Data analysis

Statistical analyses were performed using InVivoStat (Clark *et al*., 2012) and used raw or transformed data (arcsine, log10(score+1) or square root), according to whichever optimized the homogeneity of variance in the ‘predicted versus residuals’ plot in InVivoStat. The ‘normal probability plot’ in InVivoStat was used to examine whether or not the data were normally distributed. If not, a rank transformation was applied: that is, the data were assigned ranks, as for a non-parametric analysis, but the ranks were then subjected to parametric tests.

Repeated-measures analyses were used to examine data from the 5-CSRTT. The analyses used a mixed model approach: ‘within-animal’ correlations were modelled using a compound symmetric covariance structure (which assumes sphericity of the variance/covariance matrix). ‘Genotype’ and ‘colony’ were used as between-subject factors and ‘treatment’ was the within-subject factor. A fourth factor was ‘time of day’ (i.e. AM session/PM session). Our previous studies indicated that time of testing influences behaviour in the 5-CSRTT (Yan *et al*., 2011; Weir *et al*., 2014) and so this was used as a blocking factor, to account for any additional variance in the data. This factor was collapsed across all subjects if there was no effect of time of day on a given dependent variable. A main effect of genotype or treatment, or an interaction between them, was used as the criterion for carrying out *post hoc* pairwise comparisons.

In the LDEB and EPM, two-way anovas were performed on raw or transformed data, with the main factors genotype and treatment. First, the anova compared the factors across all groups (uninjected, vehicle and drug treated). If there was a main effect of either factor, or an interaction between them, further analyses were carried out using *post hoc* anova to compare vehicle controls with drug treatment (main effect of ‘drug’). Where there was a main effect of genotype or drug, or relevant interactions between the factors, *post hoc* LSD tests were performed to compare pairs of data.

### Materials

Guanfacine hydrochloride was purchased from Tocris (Abingdon, UK), dissolved in 0.9% saline and injected i.p. in a volume of 10 mL·kg^−1^ throughout.

## Results

Our previous experiments have confirmed that repeated experience of the VITI can influence behaviour in this test (see Weir *et al*., 2014). For this reason, the response to guanfacine in this study was compared with NI-2, which was the uninjected control embedded within the series of drug treatments. Findings from testing in NI-1 are to be reported elsewhere.

The 5-CSRTT experiment was carried out using mice derived from two colonies (homozygous breeders and heterozygous breeders). However, there was no statistically significant interaction between colony and the response to vehicle or drug treatment, for any of the dependent variables, and so the data were collapsed across colony.

As before (Yan *et al*., 2011; Weir *et al*., 2014), certain behaviours (specifically, *%omissions* and *%premature responses*) depended on time of day, which was used as a blocking factor in the analysis of these behaviours. Where there were no effects of time of day, data were collapsed across this factor.

### Guanfacine has bidirectional effects on %omissions in NK1R−/− mice (Figure 1A)

**Figure 1:**
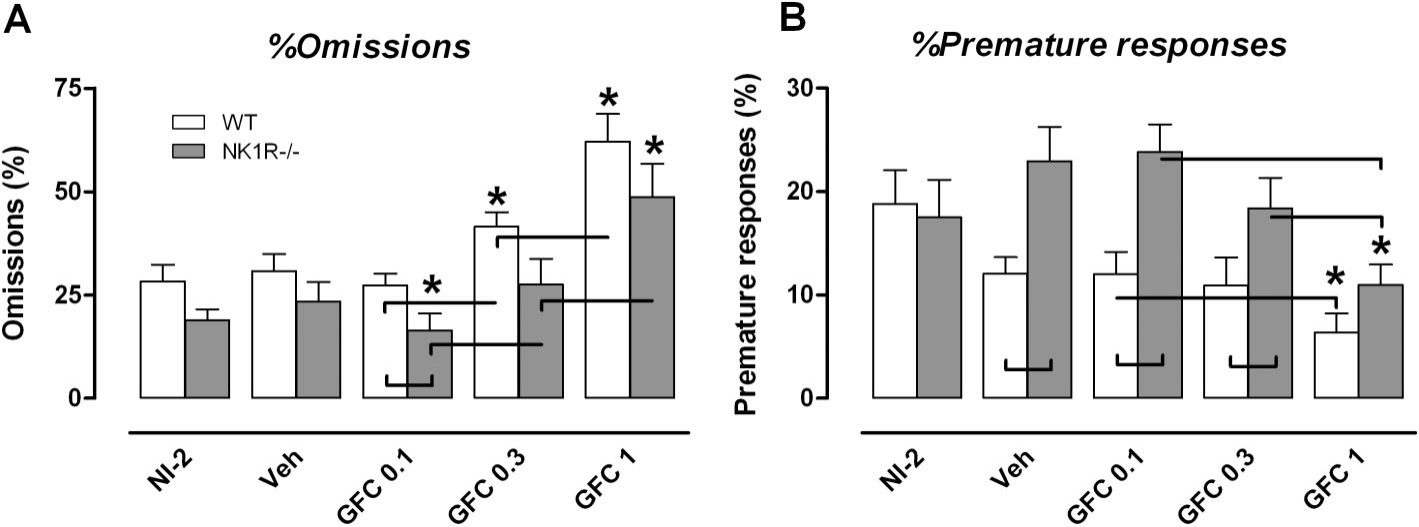
Effect of guanfacine (GFC; 0.1, 0.3 and 1.0 mg·kg^−1^, i.p.) on (A) *%omissions* and (B) *%premature responses* in the 5-CSRTT, compared with vehicle (saline), or NI-2 in wild-type (WT) and NK1R−/− mice. The lowest dose of guanfacine reduced *%omissions* in NK1R−/− mice, but not WTs. Guanfacine also decreased *%premature responses*, but to the same extent in both genotypes. Data show mean ± SEM. *N* = 9–10 per group. Lines linking bars indicate statistical significance of at least *P* < 0.05; * indicates *P* < 0.05 versus vehicle within genotype.

There were no overall differences in *%omissions* between the two genotypes [[sqrt, all groups] geno: *F*_(1,18)_ = 3.40, *P =* 0.082] (Figure 1A). The effects of guanfacine across all doses also did not depend on genotype [[sqrt, veh vs. drug] drug*geno: *F*_(3,49)_ = 1.33, *P =* 0.276]. However, this drug had bidirectional effects on this behaviour [[sqrt] drug: *F*_(3,49)_ = 48.00, *P* < 0.001]. The lowest dose (0.1 mg·kg^−1^) reduced *%omissions* in NK1R−/− mice only, by comparison with either vehicle-treated NK1R−/− mice [[sqrt] veh vs. 0.1 mg·kg^−1^: *P =* 0.004] or drug-treated WTs [[sqrt] 0.1 mg·kg^−1^, WT vs. KO: *P =* 0.049]. The highest dose of guanfacine (1.0 mg·kg^−1^) increased *%omissions* in both genotypes to a similar extent [[sqrt] veh vs. 1.0 mg·kg^−1^, WT: *P* < 0.001, KO: *P* < 0.001].

### Guanfacine attenuates premature responding in WT and NK1R−/− mice (Figure 1B)

Overall, NK1R−/− mice expressed more *%premature responses* than WTs [[sqrt, all groups] geno: *F*_(1,18)_ = 8.39, *P =* 0.010] (Figure 1B). Guanfacine reduced the incidence of this behaviour, [[sqrt, veh vs. drug] drug: *F*_(3,49)_ = 9.45, *P* < 0.001], especially at the highest dose [[sqrt] veh vs. 1.0 mg·kg^−1^, WT: *P* = 0.012, KO: *P* < 0.001]. However, the effect of the drug did not depend on genotype [[sqrt, veh vs. drug] drug*geno: *F*_(3,49)_ = 0.39, *P* = 0.759].

### Guanfacine blunts behaviour in measures of arousal and motivation (Table 2)

**Table 2:** The effects of guanfacine on behaviour of NK1R−/− and wild-type (WT) mice in the 5-CSRTT

Behaviour	WT					NK1R−/−				
	NI-2	Veh	0.1 mg·kg^−1^	0.3 mg·kg^−1^	1.0 mg·kg^−1^	NI-2	Veh	0.1 mg·kg^−1^	0.3 mg·kg^−1^	1.0 mg·kg^−1^
Total number of trials	96.0 ± 3.5	99.8 ± 0.2	99.2 ± 0.8	99.0 ± 0.8	90.9 ± 5.0*	99.3 ± 0.7	96.5 ± 1.8	100.0 ± 0	100.0 ± 0	89.1 ± 7.4
%Accuracy	96.5 ± 0.85	96.0 ± 1.41	96.1 ± 1.11	94.6 ± 1.82	92.2 ± 2.03	96.2 ± 0.82	92.6 ± 1.25	95.6 ± 0.91	94.0 ± 1.42	86.5 ± 4.20
Latency to correct hole	0.93 ± 0.06	1.01 ± 0.04	1.01 ± 0.06	1.12 ± 0.06	1.28 ± 0.11*	0.96 ± 0.06	1.01 ± 0.06	1.04 ± 0.07	1.10 ± 0.05	1.61 ± 0.21*
Latency to magazine	1.48 ± 0.08	1.52 ± 0.07	1.76 ± 0.07*	2.64 ± 0.37*	4.57 ± 1.01*	1.44 ± 0.09	1.75 ± 0.19	1.90 ± 0.14	2.09 ± 0.26	4.11 ± 0.87*

Values show mean ± SEM. *N* = 9–10 per group.

* *P* < 0.05 versus vehicle control (highlighted in bold).

*Accuracy*, *total trials*, *latency to correct response* and *latency to the magazine* were all modified by guanfacine to the same extent in both genotypes (Table 2). Whereas guanfacine decreased *%accuracy* [[arcsine, veh vs. drug] drug: *F*_(3,49)_ = 3.57, *P =* 0.020] and *total trials* [[arcsine, veh vs. drug] drug: *F*_(3,49)_ = 3.84, *P =* 0.015], *latency to correct* [[log10, veh vs. drug] drug: *F*_(3,49)_ = 16.47, *P* < 0.001] and *latency to magazine* [[rank, veh vs. drug] drug: *F*_(3,49)_ = 39.42, *P* < 0.001] were both increased by the drug.

### Guanfacine reduces activity in the light/dark exploration box (Figure 2)

**Figure 2:**
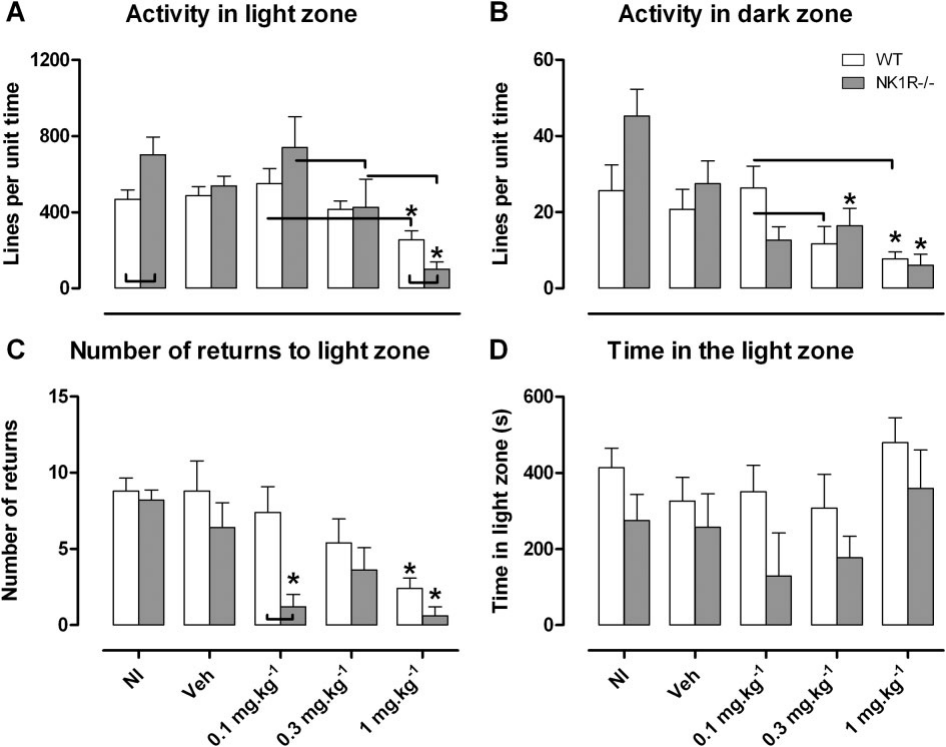
Guanfacine (0.1, 0.3 and 1.0 mg·kg^−1^, i.p.) reduced activity of wild-type (WT) and NK1R−/− mice in both the light zone and dark zone, and reduced crossings between the zones but had no effect on the time spent in either zone. (A) Activity per unit time in the light zone; (B) activity per unit time in the dark zone; (C) number of returns to the light zone; and (D) time in the light zone of a light–dark exploration box. NI, no injection; Veh, vehicle (saline). Data show mean ± SEM, *N* = 5 per group. Lines linking bars indicate statistical significance of at least *P* < 0.05 and * indicates *P* < 0.05 versus vehicle within genotype.

Compared with untreated WTs, NK1R−/− mice were hyperactive in the LZ of the LDEB [[sqrt, NI vs. veh] geno: *F*_(1,16)_ = 4.75, *P =* 0.044; NI, WT vs. KO: *P =* 0.024, Figure 2A]. An apparent hyperactivity in the DZ just missed the criterion for statistical significance [[sqrt, NI vs. veh] geno: *F*_(1,16)_ = 3.79, *P =* 0.069, Figure 2B].

Guanfacine reduced motor activity in the LZ [[rank, veh vs. drug] drug: *F*_(3,32)_ = 15.71, *P* < 0.001]: at the highest dose, activity was decreased in both genotypes [[rank] veh vs. 1.0 mg·kg^−1^, WT: *P =* 0.007, NK1R−/−: *P* < 0.001, Figure 2A]. The same response was seen in the DZ [[sqrt, veh vs. drug] drug: *F*_(3,30)_ = 5.07, *P =* 0.006]. However, an apparent reduction in locomotor activity in the DZ, after treatment with the 1.0 mg·kg^−1^ dose, was statistically significant in NK1R−/− mice only [[sqrt] veh vs. 1.0 mg·kg^−1^, WT: *P =* 0.068, KO: *P =* 0.002; Figure 2B]. Guanfacine also reduced the *number of returns* to the LZ in both genotypes [[sqrt, veh vs. drug] drug: *F*_(3,32)_ = 4.99, *P =* 0.006; Figure 2C]. In NK1R−/− mice, this response was evident at 0.1 and 1.0 mg·kg^−1^ [[sqrt] veh vs. 0.1 mg·kg^−1^: *P* = 0.017, veh vs. 1.0 mg·kg^−1^: *P* = 0.005] but only the 1.0 mg·kg^−1^ dose caused such a reduction in WTs [[sqrt] veh vs. 1.0 mg·kg^−1^: *P* = 0.023]. Overall, NK1R−/− mice spent less time in the LZ than WTs [[rank, all groups] geno: *F*_(1,32)_ = 4.75, *P* = 0.037] but this was unaffected by guanfacine [[rank, veh vs. drug]drug: *F*_(3,32)_ = 2.05, *P* = 0.127; Figure 2D]. The effect of guanfacine did not depend on genotype in any measure.

### NK1R−/− mice display increased anxiety-like behaviour in the EPM (Figure 3)

**Figure 3:**
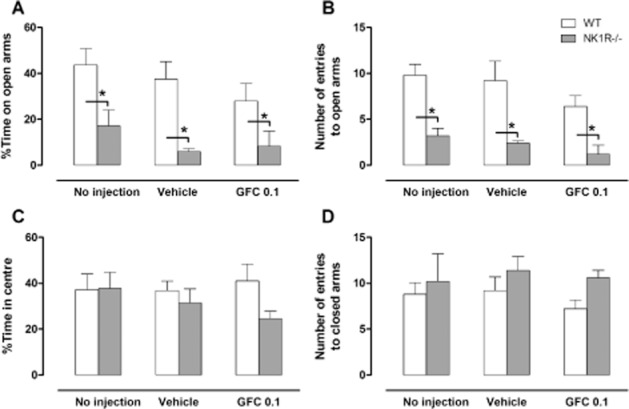
Guanfacine (GFC; 0.1 mg·kg^−1^) does not affect the behaviour of NK1R−/− mice in the EPM. (A) Time spent in the open arms; (B) number of entries to the open arms; (C) %time spent in the centre section number; and (D) number of entries to the closed arms of an EPM in wild-type (white bars) and NK1R−/− mice (grey bars). Data show mean ± SEM, *N* = 5. * *P* < 0.05.

There was a clear difference in the behaviour of NK1R−/− and WT mice in the EPM. Compared with WTs, NK1R−/− mice spent less %time on the open arms [[raw, all groups] geno: *F*_(1,24)_ = 23.38, *P* < 0.001; Figure 3A] and made fewer entries into the open arms [[rank, all groups] geno: *F*_(1,24)_ = 42.32, *P* < 0.001; Figure 3B]. There were no genotype differences in the %time spent in the centre square [[raw, all groups] geno: *F*_(1,24)_ = 2.08, *P =* 0.1621; Figure 3C], or in the number of entries to the closed arms [[sqrt, all groups] geno: *F*_(1,24)_ = 2.64 *P =* 0.1171; Figure 3D].

When compared with vehicle-treated mice, guanfacine had no overall effect on time in open arms [[raw, veh vs. drug] drug: *F*_(1,16)_ = 0.32, *P =* 0.578; Figure 3A], or the number of entries to the open arms [[rank, veh vs. drug] drug: *F*_(1,16)_ = 2.32, *P =* 0.147; Figure 3C]. The effect of guanfacine in the two genotypes did not differ for either measure.

## Discussion

The findings from this study suggest that, regardless of colony, guanfacine improves attention and reduces impulsivity in NK1R−/− mice. A lack of any statistical interaction between the factors ‘drug treatment’ and ‘colony’ renders it unlikely that a difference in the response of the two colonies is masking any effects of guanfacine on the behaviours studied here.

### Guanfacine improves ADHD-like behaviours in NK1R−/− mice

#### Attention

Unlike our previous studies (Yan *et al*., 2011; Weir *et al*., 2014), NK1R−/− mice did not display an inattentive phenotype at baseline (NI-2) in this study. This could be a result of improved performance after repeated testing (see Weir *et al*., 2014), or relate to reports that individuals with ADHD do not consistently present with the same behavioural subtype (Lahey *et al*., 2005; Todd *et al*., 2008). Another factor is that an increase in *%omissions* seems more robust when animals are tested with a prolonged, but fixed, inter-trial interval (LITI) than when using a variable ITI (unpublished observations). However, a previous study has confirmed that acute administration of an NK_1_ receptor antagonist (RP 67580) does aggravate inattentiveness in this test (Weir *et al*., 2014), suggesting that a lack of functional NK_1_ receptors could contribute to this behavioural abnormality.

Nevertheless, the lowest dose of guanfacine studied in the 5-CSRTT (0.1 mg·kg^−1^) caused a modest (−7%) reduction in %*omissions* (attention) in NK1R−/− mice, but not WTs. This beneficial response is consistent with the improvement in attention when spontaneously hypertensive rats (‘SHR’: a long-established rodent model of ADHD) are tested in an operant conditioning task (Sagvolden, 2006). This genotype-specific improvement in attention in NK1R−/− mice is unlikely to be explained by a change in animals’ state of arousal or motivation to respond because, at this dose, guanfacine did not affect the *latency to correct response* or the *total number of trials* completed by either genotype.

Unfortunately, it is difficult to make quantitative comparisons of the response to guanfacine in this study and in human trials because the scoring systems used in the two fields differ considerably. However, one human study has shown that when ADHD patients are tested in a choice reaction–time test after guanfacine extended release (GXR) treatment, they similarly show an improvement on ADHD rating scales, but no reduction in reaction speed compared with placebo (Kollins *et al*., 2011). An improved inattention subscale score has also been reported in ADHD patients after GXR treatment when given chronically (Biederman *et al*., 2008; Sallee *et al*., 2009; 2012; Newcorn *et al*., 2013) or in combination with a psychostimulant (Wilens *et al*., 2012).

This genotype-dependent response to guanfacine is interesting because it suggests that NK1R−/− mice are more sensitive to the effects of a low dose of this drug. Given the confirmed association of *TACR1* gene polymorphism(s) and ADHD, it is reasonable to predict that guanfacine could also be more effective in ADHD patients with this genetic association. Whether or not this is the case, it is not necessary for guanfacine to be effective in only this genotype in order to be an effective treatment for ADHD.

By contrast, the highest dose of guanfacine (1.0 mg·kg^−1^) increased %*omissions* (i.e. reduced attention) in both genotypes. This impairment is most likely explained by the well-documented sedative effects of this drug (Van der Laan *et al*., 1985; Jakala *et al*., 1999). This is because both the *latency to correct response* and the *latency to collect the reward* were increased, whereas the *total number of total trials* completed by the mice was reduced at this dose. An alternative explanation is that guanfacine blunted animals’ motivation to carry out the task but, to the best of our knowledge, there is no evidence that guanfacine impairs motivation. In either case, the increase in *%omissions* is unlikely to be explained by a primary effect on cognition.

In tests of vigilance and working memory, activation of α_2A_-adrenoceptors enhances performance of both rats and monkeys in delayed alternation (Carlson *et al*., 1992) and delayed response tasks (Arnsten *et al*., 1988) respectively. Conversely, depletion of cortical noradrenaline impairs sustained attentional performance in the 5-CSRTT in rats (Carli *et al*., 1983). Low doses of α_2A_-adrenoceptor agonists improve performance, particularly in animals with either depletion of cortical noradrenaline (Milstein *et al*., 2007) or in older animals showing significant cortical loss of this neurotransmitter (Arnsten *et al*., 1988). However, deficits in working memory in mice with functional ablation of α_2A_-adrenoceptors are not relieved by guanfacine (Franowicz *et al*., 2002). Noradrenergic signalling strongly influences frontal cortical regions that mediate attention and working memory (see Arnsten and Li, 2005; Robbins and Roberts, 2007) and the classical bell-shaped treatment/response curve applies (Aston-Jones and Cohen, 2005; Arnsten, 2011).

The beneficial effects of guanfacine are thought to be mediated primarily by post-synaptic α_2A_-adrenoceptors located in the prefrontal cortex (PFC), which may become supersensitive as a consequence of a long-term deficit in noradrenergic transmission (Omiya *et al*., 2008). However, guanfacine could also reduce inattentiveness by activating somatodendritic α_2A_-adrenoceptors in the locus coeruleus (LC). This nucleus, which is the sole source of noradrenaline in the PFC (Loughlin *et al*., 1982), receives inputs from both GABAergic and glutamatergic projection neurones, from the nucleus prepositus hypoglossi and nucleus paragigantocellularis respectively (Ennis and Aston-Jones, 1989; Aston-Jones *et al*., 1991). Whereas GABAergic neurones tonically inhibit LC neurones, glutamate triggers their burst spiking in response to sensory stimuli (Foote *et al*., 1980; Ennis and Aston-Jones, 1989; Kawahara *et al*., 1999). Antagonism or functional ablation of NK_1_ receptors blunts this GABAergic inhibition (Maubach *et al*., 2002; Ebner and Singewald, 2007): such disinhibition would disrupt attention (Yan *et al*., 2009) but could be prevented by activation of somatodendritic α_2A_-adrenceptors, which suppresses LC excitation.

*Accuracy* is arguably an alternative index of attention (Robbins, 2002), but there are disparate reports on the effects of guanfacine on this measure. Whereas *accuracy* was increased in one preclinical study of aged macaques (O’Neill *et al*., 2000), there was no such response in a human study of cognitive performance (Jakala *et al*., 1999). Here, *accuracy* was not affected by any dose of guanfacine in either genotype, probably because the performance of the animals in the 5-CSRTT was already near maximum for this measure (∼96%) before drug treatment. One limitation of these findings is that such a high level of accuracy prevents this from being a useful index of attention when assessing the efficacy of the low dose of guanfacine. However, it is unlikely that the improvement in *%omissions* at the low dose is explained by increased motivation because neither measure of motivation (*latency to correct response/reward*) was affected.

#### Impulsivity

As in our previous studies, NK1R−/− mice were more impulsive than WTs (Yan *et al*., 2011; Dudley *et al*., 2013; Weir *et al*., 2014) but, here, this was evident only after they had experienced an i.p. injection. The reason for this is not clear but NK_1_ receptors are known to influence the noradrenergic stress response (for reviews, see Ebner and Singewald, 2006; Stanford, 2014). Guanfacine decreased impulsivity in both WT and NK1R−/− mice: evidently, this improvement (at 1.0 mg·kg^−1^) does not depend on functional NK_1_ receptors but could be secondary to non-specific inhibition of motor behaviour. Nevertheless, such a non-specific response could also explain the efficacy of this drug in the clinic. If so, it is possible that motor impulsivity is more likely to be attenuated than impulsive choice, such as the considered choosing of small, immediate rewards over larger, delayed rewards (Bari and Robbins, 2013).

Our findings are supported by evidence from other rodent studies: for instance, a high dose of guanfacine decreased impulsivity of both high-impulsive and low-impulsive rats in the 5-CSRTT, but also increased inattentiveness and response latencies (Fernando *et al*., 2012). Similarly, the response control of rats in the stop signal reaction–time task was improved by guanfacine, but this response was accompanied by a reduction in the speed of reaction in the task (Bari *et al*., 2009). In contrast, it was recently reported that local infusion of guanfacine into the ventral hippocampus of rats caused an improvement in impulsive choice, without affecting response latencies (Abela and Chudasama, 2014).

#### Hyperactivity

As before, uninjected NK1R−/− mice were hyperactive in the LDEB by comparison with WTs (Herpfer *et al*., 2005; Fisher *et al*., 2007; Yan *et al*., 2010). This is consistent with findings from our previous study in which acute administration of the NK_1_ receptor antagonists L 733060 or RP 67580 increased locomotor activity of wild-type mice (Yan *et al*., 2010). This latter finding points to a lack of functional NK_1_ receptors as a factor that underlies this behavioural abnormality.

Guanfacine reduced locomotor activity in both genotypes and so, like impulsivity, functional NK_1_ receptors are not necessary for this response, and both are likely attributed to the sedative effects of the drug (reviewed by Scheinin *et al*., 1989). However, the greater reduction in activity and number of returns to the LZ in NK1R−/− mice suggests that these mice could be more sensitive to the actions of guanfacine.

In the clinic, somnolence, sedation and fatigue are the most frequent reasons for discontinuing guanfacine therapy (Faraone *et al*., 2013; Hirota *et al*., 2014). Notwithstanding the confounding effects of sedation on measures of impulsivity, numerous preclinical studies have dissociated the sedative and cognitive effects of α_2_-adrenoceptor agonists (Arnsten *et al*., 1988; Franowicz and Arnsten, 1998; Jakala *et al*., 1999). For instance, the spatial working memory of rhesus monkeys was improved at a dose of guanfacine that had no sedative or hypotensive effects (Arnsten *et al*., 1988).

### NK1R−/− mice display an anxiogenic phenotype

Anxiety is a common co-morbid problem for ADHD patients (Sobanski, 2006), and the two disorders may interact. For instance, Mancini *et al*. (1999) reported that the age of onset of anxiety is earlier, and its severity greater, in patients who had experienced childhood ADHD. Furthermore, ADHD patients with co-morbid anxiety have more pronounced attentional deficits (Sobanski, 2006).

Although the LZ of the LDEB was configured so as to render it ‘novel’, rather than aversive (i.e. low light intensity), these NK1R−/− mice (on a mixed background) displayed greater anxiety-like behaviour by comparison with their wild-types. This finding is consistent with our previous reports that NK1R−/− mice express greater active and passive avoidance of the LZ of the LDEB (Herpfer *et al*., 2005; Fisher *et al*., 2007). Nevertheless, the LDEB protocol used here has not been validated as a screen for anxiolytic drugs. For this reason, we also compared the behaviour of NK1R−/− and WT mice in the EPM. In this procedure, the NK1R−/− mice spent less time (∼17 vs. 44% in WTs) on the open arms and made fewer entries to the open arms, confirming that the NK1R−/− phenotype expresses more anxiety-like behaviour than WTs.

The effect of functional ablation of the NK_1_ receptor gene on anxiety-like behaviour and locomotor activity seems to depend on genetic background. This is inferred from evidence that mice lacking NK_1_ receptors on a 129/SvEv background spend a *greater* proportion of time on the open arms of an EPM than their WTs (i.e. they express less anxiety-like behaviour; Santarelli *et al*., 2001). By contrast, NK1R−/− mice on either a 129/Sv × C57BL/6 background (Murtra *et al*., 2000) or a J129/C57 hybrid background (Rupniak *et al*., 2001) do not. In fact, compared with WTs, NK1R−/− mice on a 129/Sv × C57BL/6 background spent *less* time on the EPM open arms (Gadd *et al*., 2003), an action that is consistent with increased anxiety-like behaviour. Furthermore, as we have reported previously (see above), mice from this colony display hyperactivity, compared with WTs, whereas for C57Bl6 mice, there is no such genotype difference (McCutcheon *et al*., 2008). It would be interesting to learn whether the abnormal locomotor activity and cognitive performance of NK1R−/− mice are similarly affected by background strain.

Guanfacine did not affect anxiety-like behaviour of either genotype in either the LDEB or the EPM test, at any dose. This finding contrasts with a report that this drug reduces anxiety-like behaviour of SHR, but not Wistar or WKY control rats, in the EPM (Langen and Dost, 2011). However, reports of the efficacy of guanfacine as an anxiolytic are inconsistent, despite its use for this clinical indication (Neylan *et al*., 2006; but see Connor *et al*., 2013). Nevertheless, the important finding here is that the effects of this drug on the behaviour expressed by NK1R−/− mice in the 5-CSRTT are unlikely to be secondary to a change in animals’ emotional status.

## Conclusions

The effects of guanfacine on the behaviour of NK1R−/− mice in the 5-CSRTT closely resemble its clinical profile in ADHD patients. The finding that guanfacine can improve attention, without affecting impulsivity, supports our proposal that impulsivity and attention are underpinned by different neuronal networks (Dudley *et al*., 2013; Stanford, 2014). However, this distinctive response was evident only at the lowest dose of guanfacine, suggesting that, at higher drug doses, its influence on these networks is either less selective, or that the networks interact.

Because guanfacine did not affect anxiety-like behaviour at any dose, it is unlikely that the response to this drug is confounded by an underlying difference in co-morbid anxiety in NK1R−/− mice. Nevertheless, the influence of background strain on anxiety-like behaviour implies that modulation of this abnormal behaviour rests on an interaction between the NK_1_ receptor gene and other gene(s), or epigenetic factor(s), as yet unidentified. Such an influence is potentially clinically important because anxiety is a common co-morbid problem for ADHD patients (Sobanski, 2006). In light of these findings, the possibility that *TACR1* polymorphism(s) contribute to co-morbid anxiety in ADHD patients merits further investigation.

## Abbreviations

5-CSRTT: 5-choice serial reaction–time task
ADHD: attention deficit hyperactivity disorder
DZ: dark zone
EPM: elevated plus maze
KO: knockout
LDEB: light/dark exploration box
LZ: light zone
VITI: variable inter-trial interval
WT: wild-type
